# Post-esophageal atresia repair double acquired tracheoesophageal fistulas treated successfully by gastric transposition: a case report

**DOI:** 10.1186/s40792-020-01004-7

**Published:** 2020-09-25

**Authors:** Nobuhiro Takahashi, Yasushi Fuchimoto, Teizaburo Mori, Kiyotomo Abe, Yohei Yamada, Goro Koinuma, Tatsuo Kuroda

**Affiliations:** 1 Department of Pediatric Surgery, Keio University School of Medicine, Tokyo, Japan; 2 Department of Pediatrics, Keio University School of Medicine, Tokyo, Japan; 3 Department of Pediatric Surgery, International University of Health and Welfare, 852 Hatakeda, Narita, Chiba Japan; 4 Pediatric Pulmonology, National Center for Child Health and Development, Tokyo, Japan

**Keywords:** Anastomotic stenosis, Esophageal atresia, Gastric transposition, Tracheoesophageal fistula

## Abstract

**Background:**

Postoperative recurrence of tracheoesophageal fistula (TEF) is a frequent complication in the repair of esophageal atresia (EA). Based on the recent etiologic classification, a TEF that develops in a different new pathway from the original one is categorized as an acquired TEF. The TEFs that fall into this category have been reported to be refractory and their mechanisms have not been fully understood. Here, we report the complicated case of an acquired TEF derived from mediastinitis after the original TEF repair developed an anastomotic stricture. The TEF contained double fistulas, both towards the right lobe bronchi, and was repaired by gastric transposition through a retrosternal route.

**Case presentation:**

The patient was diagnosed with Gross C esophageal atresia after birth and underwent tracheoesophageal fistula banding during the neonatal period. He experienced an intractable anastomotic stenosis after surgery which was treated with repeated balloon dilation therapy. By the age of 11 months, he developed a mediastinal abscess that improved with conservative treatment. At 18 months old, a fistula from the esophagus to the right superior lobe bronchus was identified. The patient underwent a right upper lobectomy to resect the fistula. However, at 21 months old, another fistula to the right lower lobe was revealed. An esophageal banding was done to relieve the respiratory symptoms. This was followed by esophagectomy and gastric transposition through the retrosternal route at 26 months old. The patient started rehabilitation and oral intake gradually after surgery. By 3 years after gastric transposition, he could already take blended food orally with the support of small amounts of enteral feeding.

**Conclusion:**

Cases of TEF derived from severe inflammation have the potential to form a complicated network and lead to recurrence. Surgeons should consider the possibility of multiple tiny fistulas in cases of severe acquired TEF. These may be repaired successfully by gastric transposition through the retrosternal route.

## Background

Postoperative recurrence of tracheoesophageal fistula (TEF), which arises in 2–11% of patients, is a frequent complication in the repair of esophageal atresia (EA) [1–6]. Reoperation against recurrent TEF is usually done with the interposition of the surrounding soft tissues such as the pleura, pericardium, and muscle flaps [2, 7]. However, up to 21% of cases, experience refistulization with mortality rates reaching approximately 4% [2, 4, 6, 8].

Recently, Smithers et al. proposed to categorize postoperative TEF into 3 groups. “Congenital TEF” is the group comprised of patients with missed or incompletely repaired TEF; “recurrent TEF” is for patients with a recurrent TEF which develops in the original location; “acquired TEF” refers to TEFs which form in a new pathway [8]. Among these three categories, acquired TEF accounts for 26% of cases and is believed to be related with an esophageal anastomotic leak (with or without a coexistent stricture). However, the mechanism of acquired TEF development is still unclear. The understanding of its pathogenesis may help the surgeon in formulating an ideal strategy against complex acquired TEF cases.

Here, we report the complicated case of an acquired TEF derived from mediastinitis after the initial TEF repair followed by multiple times of balloon dilation for an anastomotic stricture. The stricture contained double fistulas to both the right upper and lower lobe bronchi, and was successfully repaired by gastric transposition through the retrosternal route. The pathogenesis and treatment strategies against acquired TEF are discussed based on our experience.

## Case presentation

The patient was born via spontaneous vaginal delivery at 33 weeks and 6 days age of gestation with a birthweight of 1603 g. After birth, he was diagnosed with Gross C esophageal atresia and polydactyly and no coexisting heart disease. The gap of the esophagus was 2.5 vertebrae, and he underwent gastrostomy and tracheoesophageal fistula banding at 0 days and 3 days old, respectively. At 59 days old, he underwent a surgery for end-to-end anastomosis of esophagus from right thoracic approach. However, at 2 months after the surgery, he developed an intractable anastomotic stenosis and underwent balloon dilation therapy ten times with a pace of once a 3 weeks. At 11 months old (1 month after the last balloon dilation), he developed a mediastinal abscess probably due to an anastomotic rupture. Fortunately, the abscess improved with conservative treatment by antibiotics. At 18 months old, the patient experienced refractory pneumonia and consulted in another institution. He was referred to us due to the discovery of a fistula between the esophagus and the right superior lobe bronchus.

**Fig. 1:**
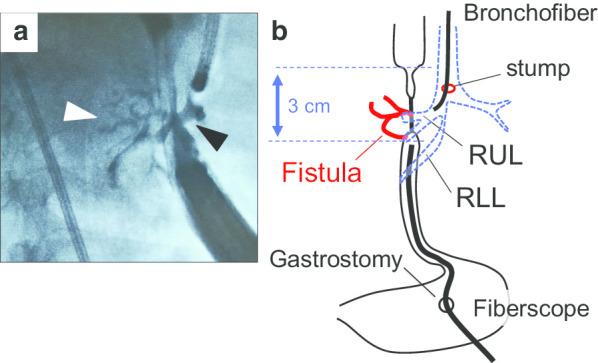
Fistula from the esophagus to the right upper lobe bronchus. **a** Contrast study before lobectomy. The contrast agent and dye are seen pouring from the fiberscope through the gastrostomy and the fistula (white arrowhead) to the right upper lobe bronchus (black arrowhead). This is confirmed by a bronchofiber. The stump of the original TEF detected in the posterior portion of the carina looks intact. **b** The scheme of the fistula. RUL: right upper lobe bronchus. RLL: right lower lobe bronchus

The patient presented with cough, and sometimes, he experienced cyanosis with percutaneous oxygen saturation (SpO_2_) levels less than 90% upon crying. These suggested a severe gastroesophageal reflux into the right bronchus with high abdominal pressure. The contrast radiography showed a fistula between the esophagus and the distal portion of the right upper lobe bronchus. This was confirmed with bronchoscopy which showed the dye pouring out from the endoscope through the gastrostomy (Fig. 1a, b). Considering this situation, we prioritized the improvement of the patient’s respiratory condition by contemplating surgery against the TEF. We expected that detection of the fistula would be difficult due to the severe adhesion caused by inflammation. We opted to perform a right upper lobectomy in the 21-month-old patient. His respiratory condition improved significantly after surgery; however, another fistula to the right inferior lobe bronchus became evident by contrast radiography 1 month after lobectomy.

When radical surgery was considered, esophagectomy and end-to-end anastomosis were thought to be impossible due to the 3 cm gap between the normal esophagus across the stenotic lesion. According to the strategy for long-gap esophageal atresia, we selected gastric transposition as the method of replacement with alternative organs. However, his stomach was assumed to be atrophic due to the long-term feeding from the transjejunal tube placed through the stenotic esophageal lesion. Feeding with a gastrostomy was required to improve this situation before surgery. To reduce the flow from the gastroesophageal reflex and switch to gastric gavage, esophageal banding and gastrostomy were performed at 23 months of age. An upper abdominal midline incision was made from the xiphoid to the umbilicus to approach the cardia. Adhesion between stomach and lateral segment of liver was dissected. The abdominal esophagus was encircled and esophageal banding was done at 4 cm above the cardia with cotton tape. At the same time, the feeding tube was replaced with a suture to keep the route of the stenotic esophageal lesion.

**Fig. 2:**
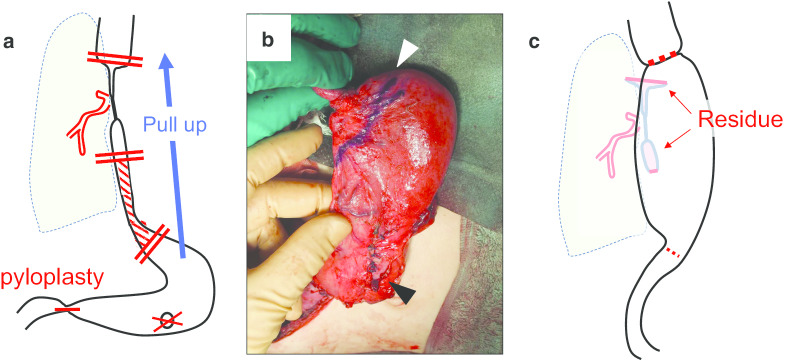
Esophagectomy and gastric transposition. **a** Scheme of operation. The cervical and abdominal portions of the esophagus are seen being mobilized and dissected. The closing of the gastrostomy and dissection of the esophagogastric junction, followed by gastric pull up with pyloroplasty, are shown. Shaded area was removed esophagus. **b** Mobilized stomach. The dissected line is shown with a white arrowhead. The closed gastrostomy is shown with a black arrowhead. **c** Scheme of postoperative design. The mobilized stomach is seen being pulled up through the retrosternal route, while the residual esophagus remains in the posterior mediastinum

At 26 months old, we performed esophagectomy and gastric transposition (Fig. 2a). The patient was placed in a supine position and the suture inserted through the stenotic site was replaced with a feeding tube as the intraoperative guide. The incision was made along the scar of previous operation. The gastrostomy was taken down and closed. The adhesion from the cardia to the esophagus was severe and difficult to dissect. The lower esophagus, about 4 cm from the esophageal hiatus, was isolated. The left gastroepiploic artery was dissected.

The neck procedure was then performed with a left cervical incision. The sternocleidomastoid muscle was retracted laterally and the esophagus was encircled. Although we had planned to reconstruct through the posterior mediastinal route, the adhesion between the esophagus and bronchus was so severe that adhesiolysis along esophagus seemed to be pose a very high risk of complications for tracheal injury. We decided to leave a portion of the esophagus retaining the severe adhesion. We switched to reconstruction through the retrosternal route. The oral and anal sides of the stenotic esophagus were dissected as much as possible and closed with a suture. For gastric transposition, the Kocher maneuver was performed to mobilize the duodenum. The gastroesophageal junction was resected. The left gastric artery was divided and ligated, and then, pyloroplasty was performed according to the Heineke–Mikulicz procedure. The mobilized stomach was marked with a suture and passed through the retrosternum under the assistance of a fiberscope to prevent rotation during the transfer (Fig. 2b). The mobilized stomach and the cervical esophagus stump were then anastomosed (Fig. 2c). A gastric tube was placed in the mobilized stomach through the anastomosis. A jejunostomy was performed for temporal enteral feeding.

The patient was placed on mechanical ventilation for 7 days postoperatively. At the 9th postoperative day, saliva was drained from the cervical anastomosis. This suggested a minor leakage which was later improved with conservative treatment by antibiotics and recombinant factor XIII administered for 2 weeks. Drainage through the gastric tube located in the mobilized stomach gradually increased to 300 ml per day. Stenosis of the pylorus due to bending was observed during the 2nd postoperative month. Several balloon dilations were performed; however, the stenosis remained. Adhesiolysis and re-pyloroplasty were performed 3 months from the previous operation. Skin closure was performed to avoid the compress of pylorus caused by high abdominal pressure. The patient was discharged during the 4th postoperative month following gastric transposition.

**Fig. 3:**
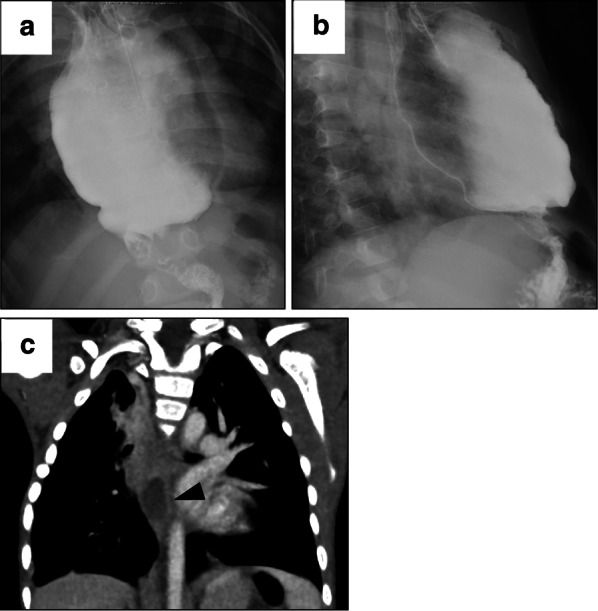
Postoperative course. **a**, **b** Frontal and lateral views of the contrast study 6 months after operation. The mobilized stomach is located midline in the mediastinum. **c** CT scan at 2 years after operation. Mucus pooling is seen in the residual esophagus (black arrowhead)

Six months after the operation, the pulled-up stomach was located midline and the mediastinum was only mildly retracted (Fig. 3a, b). The computed tomography (CT) scan taken 2 years after the operation showed a residual esophagus in the posterior mediastinum having a pool of mucus but without dilation (Fig. 3c). The patient underwent rehabilitation for food recognition and started drinking water a year after the operation. He experienced mild vomiting at night which was improved with the Fowler's position. Three years after the operation, the patient can now take blended food orally with the support of small amounts of enteral feeding through the jejunostomy.

## Discussion

We experienced a refractory case of acquired TEF with double fistula that was repaired by gastric transposition through the retrosternal route. Double fistula formation is relatively rare. The clinical course in this case suggests some aspects of acquired TEF development. The mechanisms of acquired TEF and the strategies to repair this complex case will be discussed based on the current study.

With regards to the mechanisms of acquired TEF development, this case suggests the possibility that the acquired TEF is derived from the severe inflammation. Furthermore, it may have been complicated by a network of tiny fistulas which are not always fully visualized in a contrast study. In the current case, before the right upper lobectomy was performed, the contrast study and bronchoscopy only detected a fistula to the right upper lobe bronchus. However, 1 month after lobectomy, a new fistula to right lower lobe bronchus appeared. This happened despite no adverse events in the postoperative course. Taking the improvement of the respiratory symptom after lobectomy into consideration, we assumed that several fistulas existed from the beginning and the esophageal contents only flowed into the relatively evident fistula (towards the upper lobe) due to the low pressure inside it. Therefore, the tinier fistula directed towards the right lower lobe could not be detected. Majority of acquired TEFs were reported to be related with anastomotic leakage, not only in esophageal atresia, but also in other diseases such as esophageal cancer [8, 9]. This suggests that severe inflammation derived from a leakage could form a fistula. If the original inflammation spread extensively, multiple fistulas could be formed. Possibly, some of the reported cases of “re-recurrence” might have developed through this mechanism and may have existed before the operation.

In the current case, the 3 cm stenosis of the esophagus and the severe adhesion around the anastomosis site were expected. Esophagoesophagostomy was thought to be difficult; hence, esophagus replacement was planned. However, the respiratory symptoms were so severe that we had to prioritize fistula removal by lobectomy and reduction of gastroesophageal reflex (GER) by esophageal banding. These operations, however, reduced the residual pulmonary function and shortened the available distal esophagus for esophageal replacement. There is no consensus about the optimal way for esophageal replacement, which are usually done in cases of long-gap esophageal atresia. Gastric transposition permits a good blood supply and a lower rate of leakage; however, respiratory morbidities have been reported to be slightly higher than other methods in the long term [10]. Gastric tube esophagoplasty is a relatively physiological method that minimizes the postoperative GER if it is performed with the Collis–Nissen procedure. However, it tends to have a shorter mobilized esophagus length and a higher leakage rate due to the high anastomotic tension [11–13]. Jejunal and colon interpositions are reported to have good outcomes and enough lengths of interposition. These procedures, however, need several anastomotic sites and require a precarious blood supply [14].

In the current case, aside from the long-gap esophageal atresia, two points were considered. First, the reconstruction route could have become longer if the mediastinal route could not be formed because of the severe adhesion and adopt the retrosternal route. Second, the respiratory and perioperative risks had to be avoided as much as possible. Considering these risks, we chose gastric transposition as the appropriate reconstruction method. However, we remained vigilant for the probable postoperative risks of gastric transposition such as mediastinal compression and dislocation of the pulled-up stomach into the thoracic cavity. To minimize these risks, we mobilized the stomach with the assistance of a fiberscope. Periodic X-ray tests were planned to check for stomach dislocation after the operation.

In the current case, dissection of the esophagus in the posterior mediastinal route was impossible due to severe adhesion. We chose the retrosternal route and left the residual esophagus containing stenotic sites in the posterior mediastinum. Hirschi et al. reported 41 successful cases of gastric transposition through the posterior mediastinal route, including 15 cases with a previous esophageal operation [15]. Some of these were done with a thoracotomy incision. In the current case, even if thoracotomy was added, it was considered impossible to peel off the bronchus from the surrounding tissues and the stenotic esophagus due to severe adhesions. For these reasons, the retrosternal route was adopted.

A CT scan taken 2 years after the operation showed mucus pooling in the residual esophagus. However, it was not diluted and the mucus seemed to flow into the distal bronchus through the fistula. Although there have not been any adverse events caused by the residual esophagus for 2 years since the operation, the residual esophagus is blind lesion and should be strictly followed by CT scan supported by blood test to detect the possibility of carcinogenesis, inflammation, and ballooning. As a long-term complications, some cases of esophageal cancer are reported in EA patients and routine endoscopy is recommended [16]. If an adverse event was to occur, we assumed that this would be treated by puncture drainage or esophagectomy approached from the left thoracic cavity. The current case was believed to be derived from an anastomotic rupture following balloon dilation against the esophageal anastomotic stenosis. No consensus are exist about how many times of balloon dilation are accepted; however, most of the successful cases were achieved within four sessions [17–20]. Although the majority of cases with stenotic strictures improve with balloon dilation, if the acquired TEF developed after anastomotic rupture, it may become very difficult to repair. Surgeon should keep these important points in mind when dealing with similar cases.

## Conclusion

Acquired TEFs which may develop after severe inflammation have the potential to form multiple networks of tiny fistulas and be difficult to be repair. In these cases, gastric transposition through the retrosternal route may be the appropriate treatment strategy.

## Data Availability

Data sharing is not applicable to this article, since datasets were neither generated nor analyzed for the case report.

## Abbreviations

TEF: Tracheoesophageal fistula
EA: Esophageal atresia
SpO2: Percutaneous oxygen saturation
CT: Computed tomography
GER: Gastroesophageal reflex
